# Uncovering precision phenotype-biomarker associations in traumatic brain injury using topological data analysis

**DOI:** 10.1371/journal.pone.0169490

**Published:** 2017-03-03

**Authors:** Jessica L. Nielson, Shelly R. Cooper, John K. Yue, Marco D. Sorani, Tomoo Inoue, Esther L. Yuh, Pratik Mukherjee, Tanya C. Petrossian, Jesse Paquette, Pek Y. Lum, Gunnar E. Carlsson, Mary J. Vassar, Hester F. Lingsma, Wayne A. Gordon, Alex B. Valadka, David O. Okonkwo, Geoffrey T. Manley, Adam R. Ferguson, TRACK-TBI Investigators

**Affiliations:** 1 Brain and Spinal Injury Center (BASIC), Zuckerberg San Francisco General Hospital, San Francisco, CA, United States of America; 2 Department of Neurological Surgery, Weill Institute for Neurosciences, University of California San Francisco, San Francisco, CA; 3 Department of Radiology and Biomedical Imaging, University of California San Francisco, San Francisco, CA, United States of America; 4 Ayasdi, Inc, Palo Alto, CA, United States of America; 5 Public Health, Erasmus Medical Center, Rotterdam, Netherlands; 6 Department of Rehabilitation Medicine, Icahn School of Medicine, Mount Sinai, New York, NY, United States of America; 7 Department of Neurosurgery, Virginia Commonwealth University, Richmond, VA, United States of America; 8 Department of Neurosurgery, University of Pittsburgh, Pittsburgh, PA, United States of America; 9 Department of Veterans Affairs, San Francisco VA Medical Center, San Francisco, CA, United States of America; University of Florida, UNITED STATES

## Abstract

**Background:**

Traumatic brain injury (TBI) is a complex disorder that is traditionally stratified based on clinical signs and symptoms. Recent imaging and molecular biomarker innovations provide unprecedented opportunities for improved TBI precision medicine, incorporating patho-anatomical and molecular mechanisms. Complete integration of these diverse data for TBI diagnosis and patient stratification remains an unmet challenge.

**Methods and findings:**

The Transforming Research and Clinical Knowledge in Traumatic Brain Injury (TRACK-TBI) Pilot multicenter study enrolled 586 acute TBI patients and collected diverse common data elements (TBI-CDEs) across the study population, including imaging, genetics, and clinical outcomes. We then applied topology-based data-driven discovery to identify natural subgroups of patients, based on the TBI-CDEs collected. Our hypothesis was two-fold: 1) A machine learning tool known as topological data analysis (TDA) would reveal data-driven patterns in patient outcomes to identify candidate biomarkers of recovery, and 2) TDA-identified biomarkers would significantly predict patient outcome recovery after TBI using more traditional methods of univariate statistical tests. TDA algorithms organized and mapped the data of TBI patients in multidimensional space, identifying a subset of mild TBI patients with a specific multivariate phenotype associated with unfavorable outcome at 3 and 6 months after injury. Further analyses revealed that this patient subset had high rates of post-traumatic stress disorder (PTSD), and enrichment in several distinct genetic polymorphisms associated with cellular responses to stress and DNA damage (PARP1), and in striatal dopamine processing (ANKK1, COMT, DRD2).

**Conclusions:**

TDA identified a unique diagnostic subgroup of patients with unfavorable outcome after mild TBI that were significantly predicted by the presence of specific genetic polymorphisms. Machine learning methods such as TDA may provide a robust method for patient stratification and treatment planning targeting identified biomarkers in future clinical trials in TBI patients.

**Trial Registration:**

ClinicalTrials.gov Identifier NCT01565551

## Introduction

Traumatic brain injury (TBI) annually produces 52,000 deaths, 257,000 hospitalizations and 2.2 million emergency visits in the United States (US) alone [1]. Even though TBI is a major cause of death and disability, it is currently diagnosed with crude, symptom-based tools, and few targeted treatments exist. During the initial TBI event, biomechanical forces interact with complex tissue geometry to produce nonlinear microforces, resulting in distributed multifocal lesions throughout the brain [2]. At a cellular level TBI results in membrane disruption, cell death, and diffuse axonal injury, accompanied by a cascade of secondary injury mechanisms that evolve over time [3,4]. These complex biological processes produce a poorly understood constellation of clinical symptomatology, with multifaceted impairments ranging from motor deficits to debilitating neurocognitive and personality changes. Because of the significant, multimodal heterogeneity of post-TBI symptoms, post-event treatment and follow-up pose a significant challenge. Some of these impairments may even go undiagnosed, particularly in the milder categories of TBI that include concussion.

One approach to better understand and to treat symptoms of TBI is to identify biomarkers for vulnerable patient subpopulations. However, defining clear central nervous system (CNS) biomarkers has historically been challenging, given the heterogeneity of TBI [5]. Fortunately, recent innovations in molecular biology and imaging provide unprecedented opportunities for data-rich phenotyping [6]. To aid TBI precision medicine, the National Institutes of Health-National Institute of Neurological Disorders and Stroke (NIH-NINDS) launched a major initiative to define TBI common data elements (TBI-CDEs). Hence, there is now a concerted and collaborative effort among researchers to define, collect, and analyze TBI-CDEs. The multicenter Transforming Research and Clinical Knowledge in TBI Pilot (TRACK-TBI Pilot) study provides the first prospective test of the feasibility and utility of the NINDS TBI CDEs [6,7]. As part of the effort, TRACK-TBI Pilot developed an information commons from the CDEs collected prospectively for 586 acute TBI patients from 3 level 1 trauma centers in the US.

**Fig 1:**
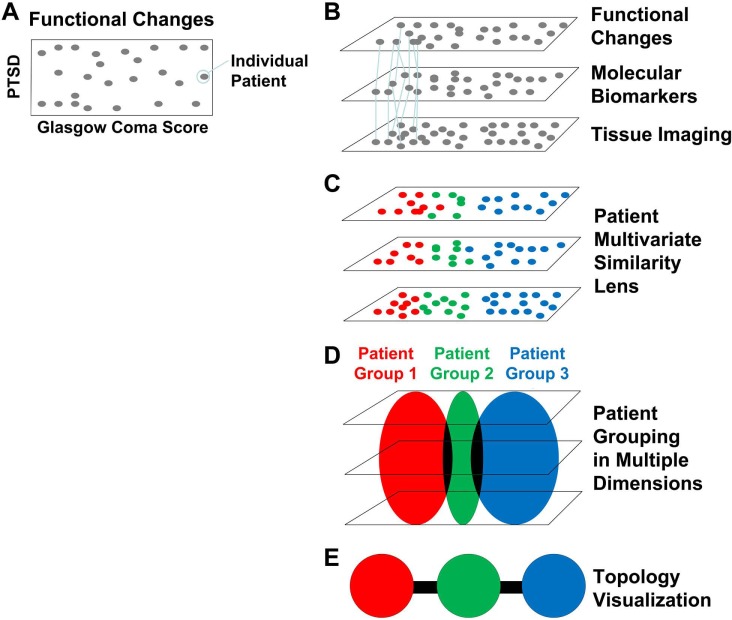
**(A-E). Methodological work-flow for integrating diverse clinical TBI data.** (A) Hypothetical example of a spatial bi-plot of individual patients (grey points) on 2 functional endpoints after TBI (GOS-E AND PTSD). The same approach can be applied to multiple metrics simultaneously using multivariate pattern detectors (e.g., principal component analysis) to produce a multivariate view of function. (B) In TRACK-TBI Pilot the same individuals (N = 586) were tracked prospectively across multiple domains (function, biomarkers, imaging) providing connections (lines) across domains to improve patient classification using the full syndromic space. (C) Multivariate pattern detection lens can be used to categorize (colors) patients across all domains. (D) Patient grouping by multivariate lens. (E) Topological visualization renders patient groups into individual nodes, colored by the multivariate lens. Edges (black lines) indicate individuals appearing in both groups producing a syndromic map of patient clusters.

Given the highly detailed and multi-scalar data in TRACK-TBI Pilot, we approached the problem from a model-free perspective, using an approach that has been developed from the application of TDA on real-world datasets [8–10]. TDA is a machine learning data analytic used to cluster patients based on functional outcome data to derive novel insights into disease mechanisms (Fig 1). To date TDA has been successfully applied to biological datasets to discover novel insights including identification of subpopulations of cancer, identification of genomic biomarkers, disease association, RNA folding, viral evolution, immunology, diabetes, and preclinical spinal cord injury and TBI[10–16]. The current study aims to test the following hypotheses in the TRACK-TBI Pilot information commons: 1) TDA will reveal data-driven patterns in patient outcomes to identify candidate biomarkers of recovery following TBI, and 2) TDA-identified biomarkers predict patient outcome recovery after TBI.

## Methods

### TBI Common Data Elements (TBI-CDEs) and the TRACK-TBI pilot study

The NIH/NINDS developed the TBI-CDEs to overcome pitfalls in TBI clinical research, including lack of standardization in data collection and analysis, inability to appropriately stratify patients, and discordant injury types [17]. Using a consensus-based approach, NINDS working groups developed standards for data capture across 4 broad domains: clinical assessments and demographic information, genetics and proteomics, neuroimaging, and outcome measures. The NINDS-CDE planning committee instructed working groups to stratify data elements into 1 of 3 categories: ‘core’, ‘basic,’ and ‘supplemental.’[18] Core elements comprise the most basic information: data that is absolutely fundamental to capture (e.g., gender, age). Basic elements provide additional diagnostic detail (e.g., education level, cause of injury). Emerging CDEs include innovative approaches that require validation before broad clinical adoption (e.g. imaging, serial plasma biomarkers).) [19]. The multicenter prospective TRACK-TBI Pilot study assessed the feasibility and utility of the TBI-CDEs in a prospective, limited multicenter (3-center) clinical observational trial [7], setting the stage for large-scale multicenter prospective efforts currently underway in the US and Europe [6,20].

### Patient enrollment

Subject eligibility was based on presentation to any of the 3 Level-1 trauma centers [(Zuckerberg San Francisco General Hospital (CA), University of Pittsburgh Medical Center (PA), and University Medical Center Brackenridge (Austin, TX)] within 24 hours of injury and a history of external force trauma to the head requiring a noncontrast head CT, in accordance with the American College of Emergency Physicians/Centers for Disease Control (ACEP/CDC) criterion [21]. Patients were excluded if pregnant, in custody, non-English speaking, or on a psychiatric hold (danger to yourself or others). Between April 2010 and May 2011 the TRACK-TBI Pilot enrolled 599 acute TBI patients; 13 subjects age <16 years were excluded due to differences in variables recommended by CDE working groups, resulting in 586 subjects in the current analysis. TRACK-TBI Pilot collected 944 raw data elements per subject. From these, a set of 213 cleaned, well-curated endpoints were distilled for meaningful analysis. Eligible subjects were enrolled through convenience sampling at all three sites. Institutional Review Board (IRB) approval was obtained at all participating sites prior to study initiation. Written informed consent was obtained from all subjects prior to enrollment in the study. For patients unable to provide consent due to the severity of their injury, consent was obtained from their legally authorized representative (LAR). Patients were then re-consented, if cognitively able, at later inpatient and/or outpatient follow-up assessments for continued participation in the study. Children aged 13 and above provided their own written consent in addition to written parental/guardian consent. Clinical characteristics for patients included in the study are summarized in Table 1.

**Table 1:** Patient clinical characteristics.

Patient Characteristics	N (%)	
(N = 586)		
Age (mean & standard deviation)	43.3 +/- 18.5	
Sex		
Female	167 (28.5%)	
Race		
White	491 (71.5%)	
Education		
Below high school	68 (12.3%)	
High school graduate	320 (57.7%)	
Bachelor's and above	167 (30.1%)	
Psychiatric History		
Present	170 (29.0%)	
Previous TBI		
No	292 (52.8%)	
Yes without hospitalization	103 (18.6%)	
Yes with hospitalization	158 (28.6%)	
Cause of Injury		
Motor vehicle accident	105 (18.0%)	
MCC/bike accident	108 (18.5%)	
Pedestrian hit	44 (7.5%)	
Fall	199 (34.1%)	
Assault	94 (16.1%)	
Other	33 (5.7%)	
ED admission GCS		
Severe (3–8)	42 (7.6%)	
Moderate (9–12)	28 (5.1%)	
Mild (13–15)	480 (87.3%)	
ED admission head CT		
Positive	259 (44.2%)	

**Abbreviations:** TBI = traumatic brain injury, MCC = motorcycle, ED = emergency department, GCS = Glasgow Coma Scale, CT = computed tomography.

### Clinical assessments and demographics

The CDE working group defined subject characteristics (i.e., demographics and social status), subject and family history, injury- or disease-related events (e.g., mechanism of injury, secondary insults), and assessments and evaluations (e.g., vital signs, intracranial pressure). The working group created a basic, intermediate, and advanced adaptation of each data element, offering investigators flexibility in the level of detail appropriate to a given study [22]. From the CDEs, the TRACK-TBI Pilot collected a combination of core, supplemental, and emerging variables.

### Genetic material

DNA and acute plasma samples (<24 hours) were collected using standardized protocols developed by the NINDS TBI CDE biospecimens and biomarkers working group [23]. TRACK-TBI Pilot also followed the meticulous guidelines regarding how samples should be obtained, processed, stored locally, stored centrally, and shipped [23].

### Neuroimaging

The NINDS neuroimaging working group supplied pathoanatomical definitions for 23 distinct lesion types to be used with any imaging modality. Core variables were distinguished as the presence or absence of individual lesions; lesion location and volumetric properties comprised most of the supplemental category, and emergent elements encompassed lesion-specific complexities. The imaging working group also provided recommendations for protocols to be used for both CT and MRI [24,25]. A board-certified neuroradiologist examined and coded all levels of neuroimaging variables. Recommended imaging parameters were implemented at all sites.

### Outcomes

The outcomes working group delineated 12 domains of behavioral outcomes. Choosing 1 measure from 11 of the 12 domains, TRACK-TBI Pilot included a broad outcomes battery. Global outcome was assessed using a standard endpoint, the Glasgow Outcome Scale-Extended (GOS-E). The GOS-E is an 8-point clinical grading scheme for categorizing the outcome and disability spectrum from ‘dead’ (GOS-E = 1), lower moderate disability (GOSE = 5), to upper good recovery (GOS-E = 8). Recovery from TBI is evidenced by achieving a higher GOS-E over time. Supplemental cognitive and psychological assessments added to a more comprehensive understanding of a domain, whereas tools in the last stages of validation were considered emerging [26]. TRACK-TBI Pilot administered the core and a subset of supplemental measures 3-, and 6-months after injury. Study personnel received *a priori* training to ensure standardization.

### Topological Data Aalysis (TDA)

TDA was performed using a cloud-based analytic platform (Ayasdi, Inc. v 3.0) on 586 patients enrolled in the TRACK-TBI pilot clinical observational trial. Patients were prospectively measured on over 900 separate variables, including the NIH/NINDs common data elements (CDEs). TDA was applied to extract the fundamental outcome features across multiple clinical variables, simultaneously. For the purposes of TDA, we limited our analysis to 17 CDEs based on their clinical importance (Table 2). These 17 CDEs included CT findings, PTSD diagnosis, and cognitive measures of processing speed and verbal learning. TDA clustered patients into subgroups (nodes) based on similarity across the 17 measures, considered simultaneously as a holistic unit (Fig 1). Subgroups that share at least 1 patient in common are joined by a line (edge). The descriptive statistics of the 17 CDEs are summarized in Table 2. Missing data were only observed in the 6-month outcome variables. Determining whether there are natural sub-types within the TBI population based on these 17 CDEs presents an analytic problem that is both multi-dimensional (17 dimensions) and multi-scalar (each CDE has different range, distributional and metric features). TDA is mathematically well-suited for dealing with this complexity (see below)[8,9,11]. Simply put, TDA uses shape-based feature detection to extract the fundamental shape of the data-space. This shape is mathematically referred to as a ‘reeb graph’ and represents the manifold of the outcome data space. We refer to the mapping of the patients within the TBI-CDEs as the ‘syndromic space’ of TBI (Fig 1A–1C). We refer to the TDA network as the TBI ‘syndromic map’ of patients within the syndromic space (Fig 1D).

**Table 2:** Descriptive statistics of CDE variables included in TDA to map TBI patients into a network topology based on TBI severity.

Variables used in TDA	N	Missing	Min	Max	Mean	SD
CT Brain Pathology	586	0	0	1	0.44	0.50
Skull Fracture	586	0	0	1	0.22	0.41
Skull Base Fracture	586	0	0	1	0.11	0.31
Facial Fracture	586	0	0	1	0.17	0.38
Epidural Hematoma	586	0	0	1	0.05	0.22
Subdural Hematoma	586	0	0	1	0.26	0.44
Subarachnoid Hemorrhage	586	0	0	1	0.26	0.44
Contusion	586	0	0	1	0.24	0.43
Midline Shift	586	0	0	1	0.07	0.25
Cisternal Compression	586	0	0	1	0.12	0.33
Marshall CT Score	586	0	1	6	1.76	1.10
Rotterdam CT Score	586	0	1	6	2.45	0.83
PTSD Diagnosis at 6 months (DSM-IV)	338	248	0	1	0.24	0.43
PTSD Checklist-Civilian Version at 6 months	338	248	17	83	32.98	14.80
WAIS Processing Speed at 6 months	305	281	50	150	99.20	15.96
CVLT: Short Delay Cued Recall at 6 months	296	290	-4.0	2.5	-0.08	1.14
CVLT: Long Delay Cued Recall at 6 months	295	291	-3.5	2.5	-0.19	1.17

**Abbreviations:** CT = computed tomography, PTSD = post-traumatic stress disorder, DSM–Diagnostic and Statistical Manual of Mental Disorders, WAIS = Wechsler Adult Intelligence Scale, CVLT = California Verbal Learning Task.

TDA clustered patients using a norm correlation metric, which measures the distance between 2 points by the Pearson correlation, given by:

$$NormCorr(X,Y)=1-{r(X}^{\prime }{,Y}^{\prime })$$ [1]

Where X', Y' are the column-wise, mean-centered, and variance normalized versions of X and Y, and

$$r(X,Y)=\frac{N\;\sum_{i=1}^{N}\;X_{i}\;Y_{i}-\sum_{i=1}^{N}X_{i}\;\sum_{i=1}^{N}Y_{i}}{\sqrt{N\;\sum_{i}X_{i}^{2}-{(\sum_{i}X_{i})}^{2}}\sqrt{N\;\sum_{i}Y_{i}^{2}-{(\sum_{i}Y_{i})}^{2}}}$$ [2]

This was combined with a lens called multidimensional scaling (MDS) coordinate 1 and MDS coordinate 2. These lenses generate a factorization of the data matrix into linearly uncorrelated components, with MDS coordinate 1 representing the highest variance, and MDS coordinate 2 representing the second-highest variance. The patient data are mapped into a Euclidean space, minimizing the sum of squares error, using the distance matrix rather than the coordinates. Gower’s normalization is then applied prior to applying MDS to generate the lens values by:

$$f(X)-\underset{z}{\min}\sum_{i,j}{\left(d(X_{i},X_{j})-L_{2}(Z_{i},Z_{j})\right)}^{2}$$ [3]

TDA then resamples the MDS space millions of times in a cloud-based supercomputer, with overlapping sample bins of variable sizes to extract the shape of the data manifold. Binning size was set at a resolution of 30 and a gain of 3.0 (equalized). The resolution setting controls the number of bin partitions patients are clustered into, similar to scaling up or down on a microscope. Increasing the resolution increases the number of nodes in the analysis graph to reveal more fine structure in the syndromic space, with fewer patients per node, preserving only the strongest connections between groups of patients. Nodes that are weakly associated tend to break apart and create smaller subgroups of patients. Gain is adjusted so that most data points will appear in the same number of bins that the gain is set to. Increasing the gain increases the number of connections between nodes/groups of patients to highlight relationships within the data. Reducing the gain value will result in smaller groups of nodes and more unconnected/single nodes. Equalizing the network distributes the patients evenly across all nodes in the network.

### Single Nucleotide Polymorphism (SNP) analysis

Previous bioassays from blood samples drawn from this TRACK-TBI Pilot cohort were analyzed to assess the role of specific genetic polymorphisms on patient outcome after mild TBI with targeted, hypothesis-driven analysis of 3 SNPs associated with altered striatal dopamine levels: ANKK1 C/T (rs1800497) [27], COMT Met/Val (rs4680) [28] and DRD2 C/T rs6277 [29] genotypes. These SNPs, along with 9 additional SNPs were incorporated in the TDA dataset. These newly incorporated SNPs included 2 more genes associated with striatal dopamine levels (ANKK1 C/G rs4938016, ANKK1 A/G rs11604671), the brain-derived neurotrophic factor (BDNF) gene (A/G rs6265), serotonin 5HT2A receptor (C/T rs6311), Apolipoprotein (Apo)E-ε2 (C/T rs7412) and ApoE-ε4 (C/T rs429358), mu opioid receptor OPRM1 (A/G rs1799971), B-cell lymphoma 2 (BCL2) gene (A/G rs17759659), and the Poly (ADP-ribose) polymerase (PARP-1) gene (A/T rs3219119).

### Targeted hypothesis testing using General Linear Models (GLM)

SNPs found to be significantly enriched in the TDA-identified sub-groups of mild TBI patients exhibiting worse GOS-E outcome between 3 and 6 months and a positive diagnosis of PTSD, detected by the PTSD checklist, civilian version (PCL) a validated tool, were formally tested for their influence on poor outcome after TBI, including PARP1, COMT, DRD2 and the 3 different ANKK1 SNPs. The statistical model was designed as a repeated measures analysis of variance (ANOVA), testing the 3-way interaction between SNP, CT pathology (yes or no), and change in GOS-E over time (3 to 6 months) performed on the full dataset. Results are reported as both within-subject effects to tease out the influence of each polymorphism on GOS-E over time either with or without CT pathology, as well as between-subject effects to test main effects of each polymorphism on GOS-E pooled outcome across 3 and 6 months, either with or without CT pathology. This targeted hypothesis testing was performed in SPSS v.19 (IBM) using the general linear model command using type III sums-of-squares and a full factorial design. Significance was assessed at p < .05.

## Results

### Natural subtypes of TBI population as defined by CDEs in a TDA network

The generated TBI syndromic map consisted of multiple sub-networks comprising of 434 clusters (from 586 patients and 17 CDEs). Similar patients are grouped as a node (Fig 1D), with similarity defined topologically, and in a multivariate fashion from all the CDEs used in the analysis. Similar nodes are close together and joined by an edge (Fig 1E). In this way patient differences are graded by location across the syndromic map. The emergence of distinct subnetworks reflects distinct subpopulations of TBI patients. We statistically explored each sub-network to understand which CDEs play the most significant role in defining similarity and dissimilarity among patient sub-clusters.

**Fig 2:**
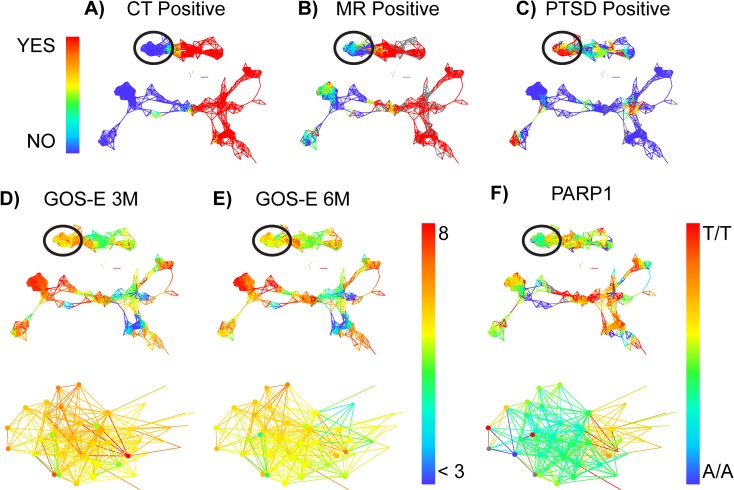
**(A-F). TBI CDE network topology identifies the PARP1 SNP as a candidate predictor of GOS-E deficits in mild TBI.** Patients with TBI were mapped into a TDA network, highlighting color schemes for CT (**A**) and MRI (**B**) pathology and whether they had a confirmed diagnosis of PTSD (DSM IV) at 6 months post-TBI (**C**). Patients in the circled regions of the network were identified due to substantial dysfunction measured by the GOS-E both at 3 months (**D**) and 6 months (**E**) post-TBI, compared with other patients in the network with no CT pathology and no diagnosis of PTSD. Data-driven exploration of these patients in the network revealed a significant categorical enrichment for the PARP1 SNP (**F**), particularly the heterozygous allele (A/T). Heat map represents range of numerical values for each measure: Panels A-C yes (1 = red) vs, no (0 = blue); Panels D-E GOS-E range from less than 3 (blue) to 8 (red); Panel F PARP1 allele A/A = 1 = blue, A/T = 2 = yellow/green, T/T = 3 = red.

The TBI syndromic map reveals that patients with acute pathological findings on CT (Fig 2A) and MR (Fig 2B) scans belonged to the same sub-networks, indicating that CDEs used in the analysis were able to cluster more severely injured TBI subpopulations together (red nodes on right half of network). On the other hand, the left sides of the connected sub-networks contained patients that were CT-negative and mostly MR-negative. (Fig 2A and 2B, blue clusters). The TBI syndromic map revealed relationships between patients as defined by the CDEs in a continuously-graded manner across multiple dimensions including Glasgow Coma Score (GCS), the Marshall CT score [30], Rotterdam CT score [31], and the presence of individual CT features, both categorical and quantitative. In addition, a clear CT-negative sub-network emerged with corresponding high GCS, indicating mild TBI (data not shown).

### Mapping of TBI severity and long-term clinical outcome measures

Fig 2 shows the network of patients clustered on the 17 CDEs using TDA. Color schemes represent the range of values for the labeled measure, including the presence of CT positive findings (Fig 2A), MRI positive findings (Fig 2B), a positive diagnosis of PTSD according to DSM IV criteria (Fig 2C). Red nodes and connections in the network highlight positive findings for these measures, showing a clear distinction between the left (blue) and the right (red) portions of the network. Our initial observation showed that the majority of patients with a diagnosis of PTSD did not show substantial brain pathology measured by either CT or MRI. When the network was colored by the GOS-E at both 3 months (Fig 2D) and 6 months (Fig 2E) after TBI, these patients with a positive PTSD diagnosis and no obvious brain pathology (N = 19) did show substantial functional deficits compared to the other CT-/MR- patients (N = 43) (circled area of the network). Data-driven exploration of this region of the network revealed a significant enrichment of the PARP1 SNP (Fig 2F) measured in these patients, not previously reported by the TRACK-TBI Pilot investigators. Results from previously identified genetic polymorphisms for ANKK1 [27], COMT [28] and DRD2 [29] were confirmed to have an impact on outcome deficits in patients with TBI (Figures in S1–S3 Figs, Tables in S1–S6 Tables).

**Fig 3:**
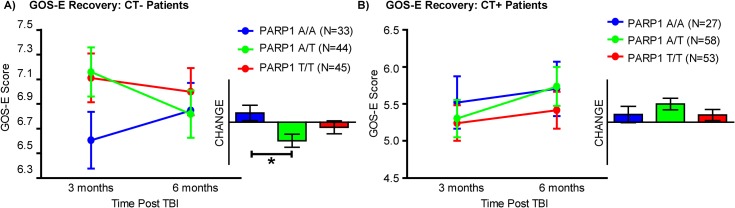
**(A-B). Hypothesis testing of PARP1 genetic polymorphism influence on GOS-E deficits in mild TBI.** GOS-E scores between 3 and 6 months post-TBI were plotted for patients who were CT negative (**A**) or CT positive (**B**), based on the SNP allele expressed (A/A = blue, A/T = yellow/green, T/T = red). Hypothesis testing of the interaction between CT pathology and the SNP allele over time revealed a significant 3-way interaction; however, no significance was detected at each time point individually. Only change in GOS-E over time was significant in patients with a negative head CT.*p < .05.

In order to formally test the hypothesis that the PARP1 SNP was a significant predictor of GOS-E recovery in patients with mild TBI, we performed an independent analysis on the full dataset using a 3-way mixed general linear model with repeated measures. This analysis was structured as a balanced factorial design testing the impact of the following factors on GOS-E recovery: Time (repeated measure; 3 vs. 6 months), CT findings (between-subjects; yes/no) and PARP1 genotype (between subjects: AA, AT, TT). Significant between-subject effects were detected in the 3-way analysis: time by CT by PARP1 genotype interaction (N = 122 patients, PARP1 A/A (n = 33), A/T (n = 44), T/T (n = 45), p = 0.019). Patients with the T/T and A/T genotypes performed worse over time on the GOS-E compared with patients with the A/A genotype in the patients with no CT pathology (Fig 3, Tables 3 and 4). Clinical characteristics of patients in the TDA-selected subgroup circled in Fig 2 (N = 37) are summarized in Table 5, alongside clinical characteristics for all patients with data collected and analyzed for the PARP1 SNP (N = 298). The TDA-selected patient group was slightly younger (41.1 ± 14.2 TDA group, vs 43.5 ± 18.2 all PARP1 group), with 6.1% fewer females, 22.4% fewer Caucasians, and roughly 6% less likely to have finished high school or college. TDA selected patients also had 15% less previous psychiatric history, however were more likely to have a previous TBI, either with (28.9%) or without hospitalization (9.2%), and were 22% more likely to have received their TBI from an assault.

**Table 3:** General linear model statistics for PARP1 SNP interaction with CT pathology on GOS-E recovery.

CT Pathology x SNP Interactions															
Source	GOSE Score (3M)					GOSE Score (6M)					GOSE Score (3M to 6M Change)				
	SS	df	MS	F	Sig.	SS	df	MS	F	Sig.	SS	df	MS	F	Sig.
PARP1 (rs3219119)	.19	2	.09	.03	.97	3.16	2	1.58	.45	.64	1.80	2	.90	.81	.45
CT Pathology x PARP1 (rs3219119)	3.66	2	1.83	.54	.58	11.00	2	5.50	1.57	.21	8.94	2	4.47	4.03	*.02
Multiple Comparisons (Tukey HSD posthoc test)	A/A vs A/T				NT	A/A vs A/T				NT	A/A vs A/T				0.47
	A/A vs T/T				NT	A/A vs T/T				NT	A/A vs T/T				0.57
	A/T vs A/A				NT	A/T vs A/A				NT	A/T vs A/A				0.47
	A/T vs T/T				NT	A/T vs T/T				NT	A/T vs T/T				0.98
	T/T vs A/A				NT	T/T vs A/A				NT	T/T vs A/A				0.57
	T/T vs A/T				NT	T/T vs A/T				NT	T/T vs A/T				0.98

**Abbreviations:** SS = Type III Sum of Squares, df = degrees of freedom, MS = mean square, NT = not tested,

* = statistical significance.

**Table 4:** General linear model statistics for PARP1 SNP interaction on GOS-E recovery by presence or absence of CT pathology.

CT Negative																CT Positive															
Source	GOSE Score (3M)					GOSE Score (6M)					GOSE Score (3M to 6M Change)					Source	GOSE Score (3M)					GOSE Score (6M)					GOSE Score (3M to 6M Change)				
	SS	df	MS	F	Sig.	SS	df	MS	F	Sig.	SS	df	MS	F	Sig.		SS	df	MS	F	Sig.	SS	df	MS	F	Sig.	SS	df	MS	F	Sig.
PARP1 (rs3219119)	2.38	2	1.19	.54	.58	7.64	2	3.82	1.65	.20	9.24	2	4.62	3.84	*0.02	PARP1 (rs3219119)	1.44	2	.72	.16	.85	6.76	2	3.38	.71	.50	2.17	2	1.08	1.07	.35
Multiple Comparisons (Tukey HSD posthoc test)	A/A vs A/T				NT	A/A vs A/T				NT	A/A vs A/T				*0.02	Multiple Comparisons (Tukey HSD posthoc test)	A/A vs A/T				NT	A/A vs A/T				NT	A/A vs A/T				NT
	A/A vs T/T				NT	A/A vs T/T				NT	A/A vs T/T				0.33		A/A vs T/T				NT	A/A vs T/T				NT	A/A vs T/T				NT
	A/T vs A/A				NT	A/T vs A/A				NT	A/T vs A/A				*0.02		A/T vs A/A				NT	A/T vs A/A				NT	A/T vs A/A				NT
	A/T vs T/T				NT	A/T vs T/T				NT	A/T vs T/T				0.31		A/T vs T/T				NT	A/T vs T/T				NT	A/T vs T/T				NT
	T/T vs A/A				NT	T/T vs A/A				NT	T/T vs A/A				0.33		T/T vs A/A				NT	T/T vs A/A				NT	T/T vs A/A				NT
	T/T vs A/T				NT	T/T vs A/T				NT	T/T vs A/T				0.31		T/T vs A/T				NT	T/T vs A/T				NT	T/T vs A/T				NT

**Abbreviations:** SS = Type III Sum of Squares, df = degrees of freedom, MS = mean square, NT = not tested,

* = statistical significance.

**Table 5:** Clinical characteristics of TDA-identified patient subgroup with PARP1 SNP (N = 37) alongside to all patients with PARP1 SNP (N = 298).

Patient Characteristics	All PARP1 Patients (N = 298)	TDA Subgroup (N = 37)
Age (mean & standard deviation)	43.5 +/- 18.2	41.1 +/- 14.2
Sex		
Female	91 (30.5%)	9 (24.4%)
Race		
White	252 (84.6%)	23 (62.2%)
Education		
Below high school	27 (9.1%)	9 (25%)
High school graduate	167 (56.0%)	18 (50%)
Bachelor's and above	93 (31.2%)	9 (25%)
Psychiatric History		
Present	93 (31.2%)	6 (16.2%)
Previous TBI		
No	152 (51.0%)	6 (16.2%)
Yes without hospitalization	53 (17.8%)	10 (27.0%)
Yes with hospitalization	83 (27.9%)	21 (56.8%)
Cause of Injury		
Motor vehicle accident	50 (16.8%)	3 (8.1%)
MCC/bike accident	55 (18.5%)	5 (13.5%)
Pedestrian hit	24 (6.0%)	2 (5.4%)
Fall	107 (35.9%)	11 (29.7%)
Assault	47 (15.8%)	14 (37.8%)
Other	14 (4.7%)	2 (5.4%)
ED admission GCS		
Severe (3–8)	26 (8.7%)	0 (0%)
Moderate (9–12)	13 (4.4%)	0 (0%)
Mild (13–15)	230 (77.2%)	37 (100%)
ED admission head CT		
Positive	144 (48.3%)	0 (0%)
PARP1 SNP		
A/A	67 (22.5%)	9 (37.5%)
A/T	116 (38.9%)	9 (37.5%)
T/T	115 (38.6%)	6 (25%)

**Abbreviations:** PARP1 = Poly [ADP-ribose] polymerase 1, TDA = topological data analysis, TBI = traumatic brain injury, MCC = motorcycle, ED = emergency department, GCS = Glasgow Coma Scale, CT = computed tomography, SNP = single nucleotide polymorphism.

Hypothesis testing of the interaction between CT pathology and the ANKK1 SNP allele on GOS-E outcome over time revealed a significant 3-way interaction for ANKK1 Gly422Arg (rs4938016) only, and a significant difference in GOS-E scores at both 3 and 6 months for patients with a positive head CT for ANKK1 Gly318Arg (rs11604671). However, these differences were not found to significantly change over time (Figure in S1 Fig, Tables in S1 and S2 Tables). Hypothesis testing of the interaction between CT pathology and the COMT SNP allele on GOS-E outcome over time revealed both a significant influence of COMT on GOS-E recovery over time, and a 3-way interaction of GOS-E recovery time with the SNP allele and presence/absence of CT pathology, specifically in patients with a negative head CT (Figure in S2 Fig, Tables in S3 and S4 Tables). Hypothesis testing of the interaction between CT pathology and the DRD2 SNP allele on GOS-E outcome over time revealed a significant influence of DRD2 on GOS-E at 3- and 6-months post TBI; however, this was only detected in patients with a positive head CT and did not significantly change over time (Figure in S3 Fig, Tables in S5 and S6 Tables).

TDA uncovered a subgroup of mild TBI individuals with poorer outcome, associated with increased PTSD rates and specific single-nucleotide polymorphisms (SNPs) associated with DNA damage and brain dopamine processing. The results provide proof-of-concept for application of multi-scalar big-data analytics to improve TBI precision medicine

## Discussion

TDA applied to data from multiple CT and MR imaging and neuropsychological domains captured the multidimensional locus of individual patients within the TBI syndromic space. Rapid mapping of TBI outcome onto the TDA-syndromic space revealed that mild TBI can be stratified into multiple subgroups that have differentiated outcome. A large subpopulation of mild TBI subjects showed poor recovery and tendency to deteriorate from 3–6 months post-injury (Fig 2D and 2E). These same individuals had very high rates of PTSD (Fig 2C) and significant enrichment in the heterozygous allele of the PARP1 SNPs (Fig 2F) that is associated with cellular responses to stress and DNA damage [32,33].

TDA improves upon traditional outcome-prediction approaches for TBI that have relied on regression modeling of multiple predictors with respect to a single ‘gold-standard’ outcome measure (e.g., the GOS-E). By simultaneously leveraging the full information provided by all outcomes, TDA and related big-data approaches have potential to improve diagnosis and therapeutic targeting. For example, CT features and neuropsychiatric batteries provided alternative views of injury severity within the topological syndromic map (Fig 2), and considering each of these pieces of information in isolation would provide only a limited view of the full syndrome of TBI. Therefore, once the TBI syndromic space was established using the pre-selected CDEs (Table 2), we were able to harness this full set of information for all patients to discover novel predictors of recovery following TBI, including several SNPs. The most striking genetic biomarker finding was that PARP1 predicted recovery in patients with a negative head CT, who would be considered to have a mild TBI (mTBI). Previous studies have implicated PARP1 as a useful therapeutic target in humans with TBI, particularly in patients with severe TBI that are enriched for A/A allele [32]. Additionally, attempts to inhibit PARP1 in rat models of TBI have shown promise in helping to reduce cell death [33]. Therefore, PARP1 may be a useful biomarker in mTBI patients when considering patient trajectories and how to maximize recovery in patients presenting with this particular A/T SNP (rs3219119) of the PARP1 gene.

TDA also confirmed the influence of genes involved in dopamine processing reported previously in TRACK-TBI Pilot patients for ANKK1 [27] (Figure in S1 Fig, Tables in S1 and S2 Tables) and COMT [28] (Figure in S2 Fig, Tables S3 and S4 Tables), as well as the novel findings of an influence of the DRD2 SNP C/C allele associated with better recovery of GOS-E in patients with a positive head CT (Figure in S3 Fig, Tables in S5 and S6 Tables), however recent findings have suggested that the T/T allele may be predictive of better recovery on verbal learning tasks after correcting for injury severity [29]. These genes represent divergent molecular mechanisms that result in lowered brain dopamine signaling. ANKK1 T/T is associated with a 40% reduction in the DRD2 receptor [34], whereas the rs4680 SNP encodes for the Met158Val locus of COMT, and the G/G genotype has been associated with lower dopamine levels due to the increase in enzymatic activity [35]. Previous studies have investigated the effect of this mutation on personality traits, dubbing the resulting phenotype as “warrior” compared to its “worrier” counterpart. The “warrior” phenotype is associated with higher concentration, memory, and cognitive function with mixed reports on the ability to emotionally process stimuli. Specifically, there have been multiple studies linking the rs4680 G/G genotype with schizophrenia [36] and lower drug responsiveness for antidepressants and anti-narcoleptics [37,38]. The association of TBI outcome to these genotypes may be due to decreased dopamine levels rather than the specific biomolecular mechanism, leaving still unanswered questions regarding the inherent predisposition to outcome and drug responsiveness of individuals suffering traumatic brain injuries.

Taken together the results indicate that, COMT and PARP1 may be useful biomarkers in a clinical prediction model to determine whether patients with an initial diagnosis of a mild TBI will develop significant functional deficit as measured on the GOS-E. ANKK1 and DRD2, on the other hand, may be useful biomarkers in a clinical prediction model for severe TBI, and warrants further investigation and cross-validation in a larger patient cohort to test whether mitigating the downstream effects of these genetic variants will improve outcome following TBI.

The present findings illustrate the value of TDA for expanding upon traditional diagnostic and prognostic tools for TBI. TDA exhibits several benefits as compared with regression methods, which perform poorly with numerous inter-correlated (multi-collinear) variables. In a regression context, multi-collinearity can lead to over-fitting to a particular dataset, limiting diagnostic value for distinct patient populations. In addition, traditional multiple regression models for TBI have been constructed to explain the variance of a single ‘gold standard outcome,’ for example the GOS-E. Such approaches ignore the fact that TBI outcome is intrinsically multifaceted. The most precise patient information is captured by considering all of the domains (e.g., psychological, cognitive) of outcome simultaneously, as is possible with TDA. Finally, traditional statistical approaches are designed to maximize the variance explained (predicted) in outcome and their performance is benchmarked by assessing value added over alternative/competing models. TDA does not suffer from these limitations because it is fundamentally focused on extracting the most robust shape (persistent homology) [8,9] across multiple alternative data views through numerous dimensions, different patient clustering algorithms, and patient subpopulations. In essence, TDA provides direct visualization of the shape of multidimensional TBI, enabling rapid insight-discovery not achievable through traditional analytics.

TDA and similar integrative analytics hold great promise to further propel recent advances in the use of novel molecular biomarkers, imaging biomarkers, and psychosocial outcomes for TBI [6,7,39–41]. To develop targeted therapeutic interventions, TBI clinician-researchers face the complex task of stratifying patients based on multifaceted information, and integrating information about TBI is fundamentally a data-intensive undertaking that could benefit from the application of advanced statistical pattern-detection approaches for enhanced decision support. Through integrative analytics of TRACK-TBI Pilot and similar datasets from other CNS diseases, TDA may help realize the potential of precision medicine to rapidly and accurately classify TBI and to identify subpopulations to target with precision medicine approaches.

## Supporting information

S1 Fig ANKK1 SNP distribution in TDA network and hypothesis testing on GOS-E recovery between 3 and 6 months post-TBI. (**A**) Distribution of 3 separate ANKK1 SNPs in the TDA network. (**B**) GOS-E scores between 3 and 6 months post-TBI were plotted for patients who were either CT negative or CT positive, grouped based on the SNP allele expressed. Hypothesis testing of the interaction between CT pathology and the ANKK1 SNP allele on GOS-E outcome over time revealed a significant 3-way interaction for ANKK1 Gly422Arg (rs4938016) only, and a significant difference in GOS-E scores at both 3 and 6 months for patients with a positive head CT for ANKK1 Gly318Arg (rs11604671). However, these differences were not found to significantly change over time. *p < .05.
(TIF)

S2 Fig COMT SNP distribution in TDA network and hypothesis testing for impact on GOS-E recovery between 3 and 6 months post-TBI. (**A**) Distribution of the COMT SNP in the TDA network. (**B**) GOS-E scores at 3 and 6 months post-TBI were plotted for patients who were CT negative or CT positive, group based on the SNP allele expressed (Met/Met = blue, Met/Val = yellow/green, Val/Val = red). Hypothesis testing of the interaction between CT pathology and the COMT SNP allele on GOS-E outcome over time revealed both a significant association of COMT with GOS-E recovery over time, and a 3-way interaction of GOS-E recovery with the SNP allele and presence/absence of CT pathology, specifically in patients with negative head CT. # p < .05 compared to both groups.
(TIF)

S3 Fig DRD2 SNP distribution in TDA network and hypothesis testing on GOS-E recovery between 3 and 6 months post-TBI. (**A**) Distribution of the DRD2 SNP in the TDA network. (**B**) GOS-E scores between 3 and 6 months post-TBI were plotted for patients who were CT negative or CT positive, group based on the SNP allele expressed (C/C = blue, C/T = yellow/green, T/T = red). Hypothesis testing of the interaction between CT pathology and the DRD2 SNP allele on GOS-E recovery revealed a significant association of DRD2 with GOS-E at 3 and 6 months post TBI, however this was only detected in patients with a positive head CT and did not significantly change over time. *p < .05.
(TIF)

S1 Table General linear model statistics for ANKK1 SNP interaction with CT pathology on GOS-E recovery. (DOCX)

S2 Table General linear model statistics for ANKK1 SNP interaction on GOS-E recovery by presence or absence of CT pathology. (DOCX)

S3 Table General linear model statistics for COMT SNP interaction with CT pathology on GOS-E recovery. (DOCX)

S4 Table General linear model statistics for COMT SNP interaction on GOS-E recovery by presence or absence of CT pathology. (DOCX)

S5 Table General linear model statistics for DRD2 SNP interaction with CT pathology on GOS-E recovery. (DOCX)

S6 Table General linear model statistics for DRD2 SNP interaction on GOS-E recovery by presence or absence of CT pathology. (DOCX)

S1 Dataset Minimal dataset of variables used to generate and color the TDA network. Variables included in this minimal dataset are those described in Table 2 as well as GOS-E and selected SNPs for PARP1, ANKK1, COMT and DRD2 used for hypothesis testing. The first column of the dataset is the global unique identifier for the TRACK-TBI pilot dataset, which can be used to link to additional variables from these patients in the full dataset stored in the Federal Interagency Traumatic Brain Injury Research (FITBIR) informatics system (https://fitbir.nih.gov/) and the One Mind Portal (http://onemind.org/Our-Solutions/One-Mind-Portal). Access to the full dataset can be requested by qualified researchers through these data portals.
(XLSX)

S1 Metadata Relevant metadata for S1 Dataset to understand description and value ranges and codes for each variable used to generate and color the TDA network. Variables listed in column A of the S1 Metadata file are copied and transposed from the first row of variables in the S1 Dataset, and accompanied by definitions and value ranges and ordinal codes for each variable.
(XLSX)
