# Supplementary figures and images for: Uncovering precision phenotype-biomarker associations in traumatic brain injury using topological data analysis
Source: PLoS One. 2017 Mar 3;12(3):e0169490. doi: 10.1371/journal.pone.0169490 (PMC5336356; doi:10.1371/journal.pone.0169490)

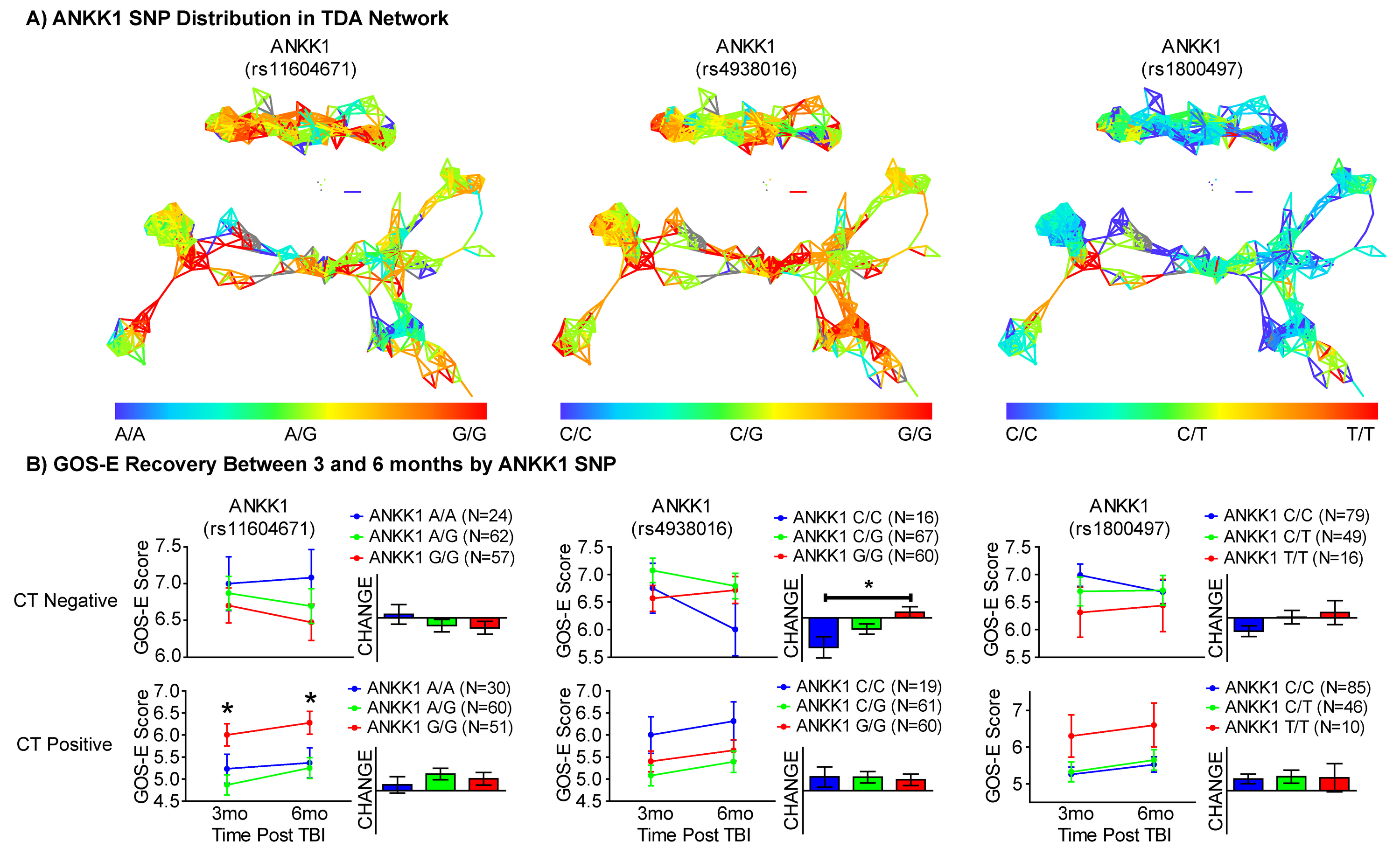

Supplement: S1 Fig — (A) Distribution of 3 separate ANKK1 SNPs in the TDA network. (B) GOS-E scores between 3 and 6 months post-TBI were plotted for patients who were either CT negative or CT positive, grouped based on the SNP allele expressed. Hypothesis testing of the interaction between CT pathology and the ANKK1 SNP allele on GOS-E outcome over time revealed a significant 3-way interaction for ANKK1 Gly422Arg (rs4938016) only, and a significant difference in GOS-E scores at both 3 and 6 months for patients with a positive head CT for ANKK1 Gly318Arg (rs11604671). However, these differences were not found to significantly change over time. *p < .05. (TIF) [file pone.0169490.s001.tif]

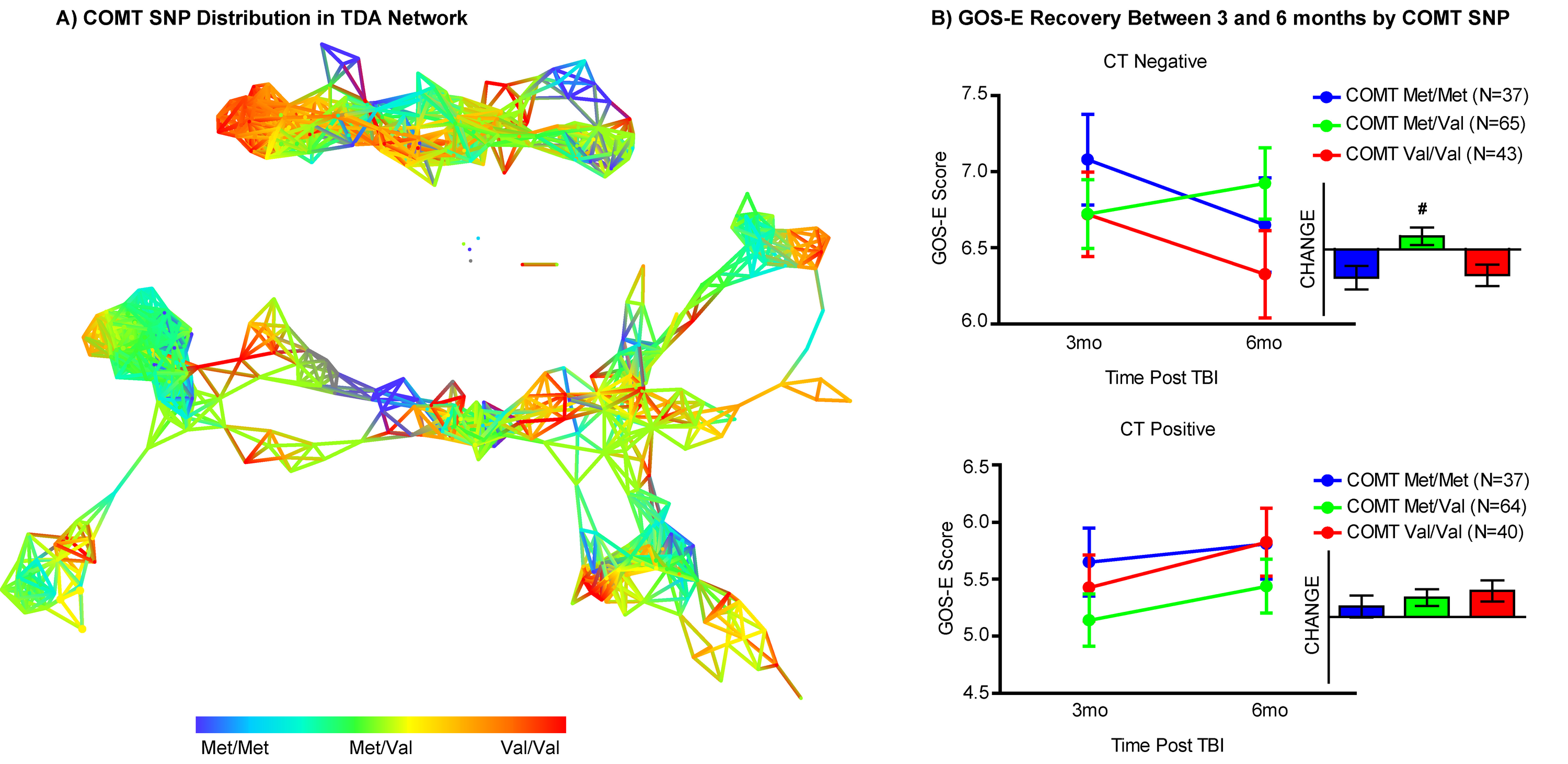

Supplement: S2 Fig — (A) Distribution of the COMT SNP in the TDA network. (B) GOS-E scores at 3 and 6 months post-TBI were plotted for patients who were CT negative or CT positive, group based on the SNP allele expressed (Met/Met = blue, Met/Val = yellow/green, Val/Val = red). Hypothesis testing of the interaction between CT pathology and the COMT SNP allele on GOS-E outcome over time revealed both a significant association of COMT with GOS-E recovery over time, and a 3-way interaction of GOS-E recovery with the SNP allele and presence/absence of CT pathology, specifically in patients with negative head CT. # p < .05 compared to both groups. (TIF) [file pone.0169490.s002.tif]

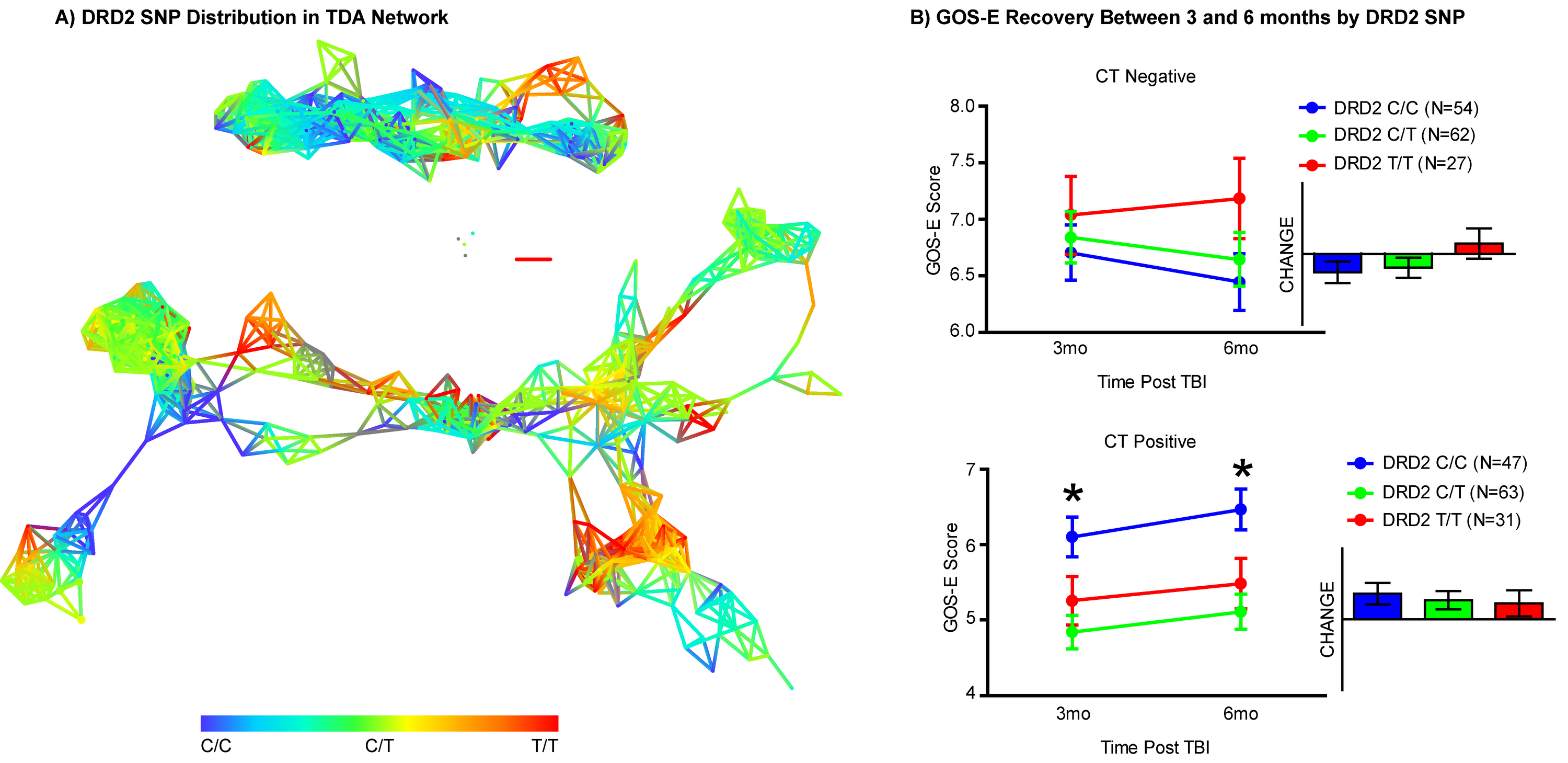

Supplement: S3 Fig — (A) Distribution of the DRD2 SNP in the TDA network. (B) GOS-E scores between 3 and 6 months post-TBI were plotted for patients who were CT negative or CT positive, group based on the SNP allele expressed (C/C = blue, C/T = yellow/green, T/T = red). Hypothesis testing of the interaction between CT pathology and the DRD2 SNP allele on GOS-E recovery revealed a significant association of DRD2 with GOS-E at 3 and 6 months post TBI, however this was only detected in patients with a positive head CT and did not significantly change over time. *p < .05. (TIF) [file pone.0169490.s003.tif]
