# Supplementary material for: Uncovering precision phenotype-biomarker associations in traumatic brain injury using topological data analysis
Source: PLoS One. 2017 Mar 3;12(3):e0169490. doi: 10.1371/journal.pone.0169490 (PMC5336356; doi:10.1371/journal.pone.0169490)
Supplement: S1 Table — (DOCX) [file pone.0169490.s004.docx]

**S1 Table. General linear model statistics for ANKK1 SNP interaction with CT pathology on GOS-E recovery.**

| **CT Pathology x SNP Interactions** | | | | | | | | | | | | | | | |
| --- | --- | --- | --- | --- | --- | --- | --- | --- | --- | --- | --- | --- | --- | --- | --- |
| **Source** | **GOSE Score (3M)** | | | | | **GOSE Score (6M)** | | | | | **GOSE Score (3M to 6M Change)** | | | | |
|  | **SS** | **df** | **MS** | **F** | **Sig.** | **SS** | **df** | **MS** | **F** | **Sig.** | **SS** | **df** | **MS** | **F** | **Sig.** |
| ANKK1 Gly318Arg (rs11604671) | 13.31 | 2 | 6.66 | 2.06 | .13 | 7.89 | 2 | 3.94 | 1.16 | .32 | .44 | 2 | .22 | .19 | .83 |
| CT Pathology x ANKK1 Gly318Arg (rs11604671) | 25.76 | 2 | 12.88 | 3.99 | ***0.01** | 28.02 | 2 | 14.01 | 4.11 | ***0.02** | 2.58 | 2 | 1.29 | 1.12 | .329 |
| Multiple Comparisons (Tukey HSD posthoc test) | A/A vs A/G | | | | .89 | A/A vs A/G | | | | .99 | A/A vs A/G | | | | NT |
|  | A/A vs G/G | | | | .47 | A/A vs G/G | | | | .49 | A/A vs G/G | | | | NT |
|  | A/G vs A/A | | | | .89 | A/G vs A/A | | | | .99 | A/G vs A/A | | | | NT |
|  | A/G vs G/G | | | | .10 | A/G vs G/G | | | | .40 | A/G vs G/G | | | | NT |
|  | G/G vs A/A | | | | .47 | G/G vs A/A | | | | .49 | G/G vs A/A | | | | NT |
|  | G/G vs A/G | | | | .10 | G/G vs A/G | | | | .40 | G/G vs A/G | | | | NT |
| ANKK1 Gly442Arg (rs4938016) | 4.14 | 2 | 2.07 | .63 | .53 | .23 | 2 | .12 | .03 | .97 | 5.29 | 2 | 2.64 | 2.36 | .10 |
| CT Pathology x ANKK1 Gly442Arg (rs4938016) | 15.96 | 2 | 7.98 | 2.43 | .09 | 19.20 | 2 | 9.60 | 2.78 | .06 | 7.64 | 2 | 3.82 | 3.40 | ***0.03** |
| Multiple Comparisons (Tukey HSD posthoc test) | C/C vs C/G | | | | NT | C/C vs C/G | | | | NT | C/C vs C/G | | | | 0.673 |
|  | C/C vs G/G | | | | NT | C/C vs G/G | | | | NT | C/C vs G/G | | | | 0.163 |
|  | C/G vs C/C | | | | NT | C/G vs C/C | | | | NT | C/G vs C/C | | | | 0.673 |
|  | C/G vs G/G | | | | NT | C/G vs G/G | | | | NT | C/G vs G/G | | | | 0.299 |
|  | G/G vs C/C | | | | NT | G/G vs C/C | | | | NT | G/G vs C/C | | | | 0.163 |
|  | G/G vs C/G | | | | NT | G/G vs C/G | | | | NT | G/G vs C/G | | | | 0.299 |
| ANKK1 Glu713Lys (rs1800497) | .73 | 2 | .37 | .12 | .89 | 4.71 | 2 | 2.36 | .68 | .51 | 2.74 | 2 | 1.37 | 1.19 | .31 |
| CT Pathology x ANKK1 Glu713Lys (rs1800497) | 17.89 | 2 | 8.94 | 2.92 | .06 | 7.96 | 2 | 3.98 | 1.14 | .32 | 1.59 | 2 | .80 | .69 | .50 |
| **Abbreviations:** SS = Type III Sum of Squares, df = degrees of freedom, MS = mean square, NT = not tested, * = statistical significance | | | | | | | | | | | | | | | |
