# Supplementary material for: Uncovering precision phenotype-biomarker associations in traumatic brain injury using topological data analysis
Source: PLoS One. 2017 Mar 3;12(3):e0169490. doi: 10.1371/journal.pone.0169490 (PMC5336356; doi:10.1371/journal.pone.0169490)
Supplement: S2 Table — (DOCX) [file pone.0169490.s005.docx]

**S2 Table. General linear model statistics for ANKK1 SNP interaction on GOS-E recovery by presence or absence of CT pathology.**

| **CT Negative** | | | | | | | | | | | | | | | | **CT Positive** | | | | | | | | | | | | | | | |
| --- | --- | --- | --- | --- | --- | --- | --- | --- | --- | --- | --- | --- | --- | --- | --- | --- | --- | --- | --- | --- | --- | --- | --- | --- | --- | --- | --- | --- | --- | --- | --- |
| **Source** | **GOSE Score (3M)** | | | | | **GOSE Score (6M)** | | | | | **GOSE Score (3M to 6M Change)** | | | | | **Source** | **GOSE Score (3M)** | | | | | **GOSE Score (6M)** | | | | | **GOSE Score (3M to 6M Change)** | | | | |
|  | **SS** | **df** | **MS** | **F** | **Sig.** | **SS** | **df** | **MS** | **F** | **Sig.** | **SS** | **df** | **MS** | **F** | **Sig.** |  | **SS** | **df** | **MS** | **F** | **Sig.** | **SS** | **df** | **MS** | **F** | **Sig.** | **SS** | **df** | **MS** | **F** | **Sig.** |
| ANKK1 Gly318Arg (rs11604671) | 1.73 | 2 | .87 | .40 | .67 | 3.87 | 2 | 1.93 | .84 | .43 | 1.70 | 2 | .85 | .68 | .51 | ANKK1 Gly442Arg (rs4938016) | 12.55 | 2 | 6.28 | 1.41 | .25 | 11.72 | 2 | 5.86 | 1.23 | .30 | .13 | 2 | .07 | .06 | .94 |
| ANKK1 Gly442Arg (rs4938016) | 8.27 | 2 | 4.13 | 1.93 | .15 | 7.68 | 2 | 3.84 | 1.69 | .19 | 12.35 | 2 | 6.18 | 5.26 | ***0.006** | ANKK1 Gly318Arg (rs11604671) | 35.97 | 2 | 17.99 | 4.20 | ***0.02** | 30.50 | 2 | 15.25 | 3.29 | ***0.04** | 1.27 | 2 | .64 | .60 | .55 |
| Multiple Comparisons (Tukey HSD posthoc test) | C/C vs C/G | | | | NT | C/C vs C/G | | | | NT | C/C vs C/G | | | | 0.27 | Multiple Comparisons (Tukey HSD posthoc test) | A/A vs A/G | | | | .71 | A/A vs A/G | | | | 1.00 | A/A vs A/G | | | | NT |
|  | C/C vs G/G | | | | NT | C/C vs G/G | | | | NT | C/C vs G/G | | | | ***0.01** |  | A/A vs G/G | | | | .24 | A/A vs G/G | | | | .12 | A/A vs G/G | | | | NT |
|  | C/G vs C/C | | | | NT | C/G vs C/C | | | | NT | C/G vs C/C | | | | 0.27 |  | A/G vs A/A | | | | .71 | A/G vs A/A | | | | 1.00 | A/G vs A/A | | | | NT |
|  | C/G vs G/G | | | | NT | C/G vs G/G | | | | NT | C/G vs G/G | | | | 0.07 |  | A/G vs G/G | | | | ***0.01** | A/G vs G/G | | | | .053 | A/G vs G/G | | | | NT |
|  | G/G vs C/C | | | | NT | G/G vs C/C | | | | NT | G/G vs C/C | | | | ***0.01** |  | G/G vs A/A | | | | .24 | G/G vs A/A | | | | .12 | G/G vs A/A | | | | NT |
|  | G/G vs C/G | | | | NT | G/G vs C/G | | | | NT | G/G vs C/G | | | | 0.07 |  | G/G vs A/G | | | | ***0.01** | G/G vs A/G | | | | .053 | G/G vs A/G | | | | NT |
| ANKK1 Glu713Lys (rs1800497) | 11.88 | 2 | 5.94 | 2.99 | .053 | 1.22 | 2 | .61 | .27 | .77 | 4.50 | 2 | 2.25 | 1.83 | .16 | ANKK1 Glu713Lys (rs1800497) | 7.25 | 2 | 3.62 | .85 | .43 | 9.63 | 2 | 4.81 | 1.00 | .37 | .09 | 2 | .05 | .04 | .96 |
| **Abbreviations:** SS = Type III Sum of Squares, df = degrees of freedom, MS = mean square, NT = not tested, * = statistical significance | | | | | | | | | | | | | | | | | | | | | | | | | | | | | | | |
