# Supplementary material for: Uncovering precision phenotype-biomarker associations in traumatic brain injury using topological data analysis
Source: PLoS One. 2017 Mar 3;12(3):e0169490. doi: 10.1371/journal.pone.0169490 (PMC5336356; doi:10.1371/journal.pone.0169490)
Supplement: S3 Table — (DOCX) [file pone.0169490.s006.docx]

**S3 Table. General linear model statistics for COMT SNP interaction with CT pathology on GOS-E recovery.**

| **CT Pathology x SNP Interactions** | | | | | | | | | | | | | | | | |
| --- | --- | --- | --- | --- | --- | --- | --- | --- | --- | --- | --- | --- | --- | --- | --- | --- |
| **Source** | **GOSE Score (3M)** | | | | | **GOSE Score (6M)** | | | | | **GOSE Score (3M to 6M Change)** | | | | |  |
|  | **SS** | **df** | **MS** | **F** | **Sig.** | **SS** | **df** | **MS** | **F** | **Sig.** | **SS** | **df** | **MS** | **F** | **Sig.** |  |
| COMT (rs4680) | 3.21 | 2 | 1.60 | .52 | .60 | 1.22 | 2 | .61 | .18 | .84 | 7.58 | 2 | 3.79 | 3.41 | ***0.03** |  |
| CT Pathology x COMT (rs4680) | .82 | 2 | .41 | .13 | .88 | 12.72 | 2 | 6.36 | 1.84 | .16 | 6.85 | 2 | 3.42 | 3.07 | ***0.05** |  |
| Multiple Comparisons (Tukey HSD posthoc test) | Met/Met vs Met/Val | | | | NT | Met/Met vs Met/Val | | | | NT | Met/Met vs Met/Val | | | | 0.64 |  |
|  | Met/Met vs Val/Val | | | | NT | Met/Met vs Val/Val | | | | NT | Met/Met vs Val/Val | | | | 0.72 |  |
|  | Met/Val vs Met/Met | | | | NT | Met/Val vs Met/Met | | | | NT | Met/Val vs Met/Met | | | | 0.64 |  |
|  | Met/Val vs Val/Val | | | | NT | Met/Val vs Val/Val | | | | NT | Met/Val vs Val/Val | | | | 1.00 |  |
|  | Val/Val vs Met/Met | | | | NT | Val/Val vs Met/Met | | | | NT | Val/Val vs Met/Met | | | | 0.72 |  |
|  | Val/Val vs Met/Val | | | | NT | Val/Val vs Met/Val | | | | NT | Val/Val vs Met/Val | | | | 1.00 |  |
| **Abbreviations:** SS = Type III Sum of Squares, df = degrees of freedom, MS = mean square, NT = not tested, * = statistical significance | | | | | | | | | | | | | | | | |
