# Supplementary material for: Uncovering precision phenotype-biomarker associations in traumatic brain injury using topological data analysis
Source: PLoS One. 2017 Mar 3;12(3):e0169490. doi: 10.1371/journal.pone.0169490 (PMC5336356; doi:10.1371/journal.pone.0169490)
Supplement: S4 Table — (DOCX) [file pone.0169490.s007.docx]

**S4 Table. General linear model statistics for COMT SNP interaction on GOS-E recovery by presence or absence of CT pathology.**

| **CT Negative** | | | | | | | | | | | | | | | | **CT Positive** | | | | | | | | | | | | | | | |
| --- | --- | --- | --- | --- | --- | --- | --- | --- | --- | --- | --- | --- | --- | --- | --- | --- | --- | --- | --- | --- | --- | --- | --- | --- | --- | --- | --- | --- | --- | --- | --- |
| **Source** | **GOSE Score (3M)** | | | | | **GOSE Score (6M)** | | | | | **GOSE Score (3M to 6M Change)** | | | | | **Source** | **GOSE Score (3M)** | | | | | **GOSE Score (6M)** | | | | | **GOSE Score (3M to 6M Change)** | | | | |
|  | **SS** | **df** | **MS** | **F** | **Sig.** | **SS** | **df** | **MS** | **F** | **Sig.** | **SS** | **df** | **MS** | **F** | **Sig.** |  | **SS** | **df** | **MS** | **F** | **Sig.** | **SS** | **df** | **MS** | **F** | **Sig.** | **SS** | **df** | **MS** | **F** | **Sig.** |
| COMT (rs4680) | 1.22 | 2 | .61 | .30 | .74 | 10.16 | 2 | 5.08 | 2.27 | .11 | 13.48 | 2 | 6.74 | 5.77 | ***0.004** | COMT (rs4680) | 2.76 | 2 | 1.38 | .32 | .73 | 4.04 | 2 | 2.02 | .42 | .66 | 1.09 | 2 | .55 | .52 | .60 |
| Multiple Comparisons (Tukey HSD posthoc test) | Met/Met vs Met/Val | | | | NT | Met/Met vs Met/Val | | | | NT | Met/Met vs Met/Val | | | | ***0.01** | Multiple Comparisons (Tukey HSD posthoc test) | Met/Met vs Met/Val | | | | NT | Met/Met vs Met/Val | | | | NT | Met/Met vs Met/Val | | | | NT |
|  | Met/Met vs Val/Val | | | | NT | Met/Met vs Val/Val | | | | NT | Met/Met vs Val/Val | | | | 0.99 |  | Met/Met vs Val/Val | | | | NT | Met/Met vs Val/Val | | | | NT | Met/Met vs Val/Val | | | | NT |
|  | Met/Val vs Met/Met | | | | NT | Met/Val vs Met/Met | | | | NT | Met/Val vs Met/Met | | | | ***0.01** |  | Met/Val vs Met/Met | | | | NT | Met/Val vs Met/Met | | | | NT | Met/Val vs Met/Met | | | | NT |
|  | Met/Val vs Val/Val | | | | NT | Met/Val vs Val/Val | | | | NT | Met/Val vs Val/Val | | | | ***0.02** |  | Met/Val vs Val/Val | | | | NT | Met/Val vs Val/Val | | | | NT | Met/Val vs Val/Val | | | | NT |
|  | Val/Val vs Met/Met | | | | NT | Val/Val vs Met/Met | | | | NT | Val/Val vs Met/Met | | | | 0.99 |  | Val/Val vs Met/Met | | | | NT | Val/Val vs Met/Met | | | | NT | Val/Val vs Met/Met | | | | NT |
|  | Val/Val vs Met/Val | | | | NT | Val/Val vs Met/Val | | | | NT | Val/Val vs Met/Val | | | | ***0.02** |  | Val/Val vs Met/Val | | | | NT | Val/Val vs Met/Val | | | | NT | Val/Val vs Met/Val | | | | NT |
| **Abbreviations:** SS = Type III Sum of Squares, df = degrees of freedom, MS = mean square, NT = not tested, * = statistical significance | | | | | | | | | | | | | | | | | | | | | | | | | | | | | | | |
