# Supplementary material for: Uncovering precision phenotype-biomarker associations in traumatic brain injury using topological data analysis
Source: PLoS One. 2017 Mar 3;12(3):e0169490. doi: 10.1371/journal.pone.0169490 (PMC5336356; doi:10.1371/journal.pone.0169490)
Supplement: S5 Table — (DOCX) [file pone.0169490.s008.docx]

**S5 Table. General linear model statistics for DRD2 SNP interaction with CT pathology on GOS-E recovery.**

| **CT Pathology x SNP Interactions** | | | | | | | | | | | | | | | |
| --- | --- | --- | --- | --- | --- | --- | --- | --- | --- | --- | --- | --- | --- | --- | --- |
| **Source** | **GOSE Score (3M)** | | | | | **GOSE Score (6M)** | | | | | **GOSE Score (3M to 6M Change)** | | | | |
|  | **SS** | **df** | **MS** | **F** | **Sig.** | **SS** | **df** | **MS** | **F** | **Sig.** | **SS** | **df** | **MS** | **F** | **Sig.** |
| DRD2 (rs6277) | 17.93 | 2 | 8.97 | 2.80 | .06 | 20.61 | 2 | 10.31 | 3.09 | ***0.05** | .95 | 2 | .48 | .41 | .66 |
| CT Pathology x DRD2 (rs6277) | 29.19 | 2 | 14.60 | 4.56 | ***0.01** | 40.16 | 2 | 20.08 | 6.03 | ***0.003** | 2.74 | 2 | 1.37 | 1.19 | .31 |
| Multiple Comparisons (Tukey HSD posthoc test) | C/C vs C/T | | | | ***0.04** | C/C vs C/T | | | | .06 | C/C vs C/T | | | | NT |
|  | C/C vs T/T | | | | .48 | C/C vs T/T | | | | .71 | C/C vs T/T | | | | NT |
|  | C/T vs C/C | | | | ***0.04** | C/T vs C/C | | | | .06 | C/T vs C/C | | | | NT |
|  | C/T vs T/T | | | | .64 | C/T vs T/T | | | | .55 | C/T vs T/T | | | | NT |
|  | T/T vs C/C | | | | .48 | T/T vs C/C | | | | .71 | T/T vs C/C | | | | NT |
|  | T/T vs C/T | | | | .64 | T/T vs C/T | | | | .55 | T/T vs C/T | | | | NT |
| **Abbreviations:** SS = Type III Sum of Squares, df = degrees of freedom, MS = mean square, NT = not tested, * = statistical significance | | | | | | | | | | | | | | | |
