# Supplementary material for: Uncovering precision phenotype-biomarker associations in traumatic brain injury using topological data analysis
Source: PLoS One. 2017 Mar 3;12(3):e0169490. doi: 10.1371/journal.pone.0169490 (PMC5336356; doi:10.1371/journal.pone.0169490)
Supplement: S6 Table — (DOCX) [file pone.0169490.s009.docx]

**S6 Table. General linear model statistics for DRD2 SNP interaction on GOS-E recovery by presence or absence of CT pathology.**

| **CT Negative** | | | | | | | | | | | | | | | | **CT Positive** | | | | | | | | | | | | | | | |
| --- | --- | --- | --- | --- | --- | --- | --- | --- | --- | --- | --- | --- | --- | --- | --- | --- | --- | --- | --- | --- | --- | --- | --- | --- | --- | --- | --- | --- | --- | --- | --- |
| **Source** | **GOSE Score (3M)** | | | | | **GOSE Score (6M)** | | | | | **GOSE Score (3M to 6M Change)** | | | | | **Source** | **GOSE Score (3M)** | | | | | **GOSE Score (6M)** | | | | | **GOSE Score (3M to 6M Change)** | | | | |
|  | **SS** | **df** | **MS** | **F** | **Sig.** | **SS** | **df** | **MS** | **F** | **Sig.** | **SS** | **df** | **MS** | **F** | **Sig.** |  | **SS** | **df** | **MS** | **F** | **Sig.** | **SS** | **df** | **MS** | **F** | **Sig.** | **SS** | **df** | **MS** | **F** | **Sig.** |
| DRD2 (rs6277) | 2.02 | 2 | 1.01 | .46 | .63 | 9.29 | 2 | 4.65 | 2.06 | .13 | 3.16 | 2 | 1.58 | 1.28 | .28 | DRD2 (rs6277) | 43.45 | 2 | 21.73 | 5.14 | ***0.007** | 47.67 | 2 | 23.83 | 5.28 | ***0.006** | .40 | 2 | .20 | .19 | .83 |
| Multiple Comparisons (Tukey HSD posthoc test) | C/C vs C/T | | | | NT | C/C vs C/T | | | | NT | C/C vs C/T | | | | NT | Multiple Comparisons (Tukey HSD posthoc test) | C/C vs C/T | | | | ***0.005** | C/C vs C/T | | | | ***0.005** | C/C vs C/T | | | | NT |
|  | C/C vs T/T | | | | NT | C/C vs T/T | | | | NT | C/C vs T/T | | | | NT |  | C/C vs T/T | | | | .18 | C/C vs T/T | | | | .08 | C/C vs T/T | | | | NT |
|  | C/T vs C/C | | | | NT | C/T vs C/C | | | | NT | C/T vs C/C | | | | NT |  | C/T vs C/C | | | | ***0.005** | C/T vs C/C | | | | ***0.005** | C/T vs C/C | | | | NT |
|  | C/T vs T/T | | | | NT | C/T vs T/T | | | | NT | C/T vs T/T | | | | NT |  | C/T vs T/T | | | | .63 | C/T vs T/T | | | | .87 | C/T vs T/T | | | | NT |
|  | T/T vs C/C | | | | NT | T/T vs C/C | | | | NT | T/T vs C/C | | | | NT |  | T/T vs C/C | | | | .18 | T/T vs C/C | | | | .08 | T/T vs C/C | | | | NT |
|  | T/T vs C/T | | | | NT | T/T vs C/T | | | | NT | T/T vs C/T | | | | NT |  | T/T vs C/T | | | | .63 | T/T vs C/T | | | | .87 | T/T vs C/T | | | | NT |
| **Abbreviations:** SS = Type III Sum of Squares, df = degrees of freedom, MS = mean square, NT = not tested, * = statistical significance | | | | | | | | | | | | | | | | | | | | | | | | | | | | | | | |
